# Response of Mouse Visual Cortical Neurons to Electric Stimulation of the Retina

**DOI:** 10.3389/fnins.2019.00324

**Published:** 2019-04-04

**Authors:** Sang Baek Ryu, Paul Werginz, Shelley I. Fried

**Affiliations:** ^1^Boston VA Healthcare System, Boston, MA, United States; ^2^Department of Neurosurgery, Massachusetts General Hospital, Harvard Medical School, Boston, MA, United States; ^3^Institute for Analysis and Scientific Computing, Vienna University of Technology, Vienna, Austria

**Keywords:** primary visual cortex, single unit activity, electrical stimulation, extraocular stimulation, retina

## Abstract

Retinal prostheses strive to restore vision to the blind by electrically stimulating the neurons that survive the disease process. Clinical effectiveness has been limited however, and much ongoing effort is devoted toward the development of improved stimulation strategies, especially ones that better replicate physiological patterns of neural signaling. Here, to better understand the potential effectiveness of different stimulation strategies, we explore the responses of neurons in the primary visual cortex to electric stimulation of the retina. A 16-channel implantable microprobe was used to record single unit activities *in vivo* from each layer of the mouse visual cortex. Layers were identified by electrode depth as well as spontaneous rate. Cell types were classified as excitatory or inhibitory based on their spike waveform and as ON, OFF, or ON-OFF based on the polarity of their light response. After classification, electric stimulation was delivered via a wire electrode placed on the surface of cornea (extraocularly) and responses were recorded from the cortex contralateral to the stimulated eye. Responses to electric stimulation were highly similar across cell types and layers. Responses (spike counts) increased as a function of the amplitude of stimulation, and although there was some variance across cells, the sensitivity to amplitude was largely similar across all cell types. Suppression of responses was observed for pulse rates ≥3 pulses per second (PPS) but did not originate in the retina as RGC responses remained stable to rates up to 5 PPS. Low-frequency sinusoids delivered to the retina replicated the out-of-phase responses that occur naturally in ON vs. OFF RGCs. Intriguingly, out-of-phase signaling persisted in V1 neurons, suggesting key aspects of neural signaling are preserved during transmission along visual pathways. Our results describe an approach to evaluate responses of cortical neurons to electric stimulation of the retina. By examining the responses of single cells, we were able to show that some retinal stimulation strategies can indeed better match the neural signaling patterns used by the healthy visual system. Because cortical signaling is better correlated to psychophysical percepts, the ability to evaluate which strategies produce physiological-like cortical responses may help to facilitate better clinical outcomes.

## Introduction

Retinal implants provide a means to restore vision to those blinded by outer retinal degenerative diseases such as retinitis pigmentosa (RP) or age-related macular degeneration (AMRD) (Fujikado et al., 2011; Rizzo, 2011; Zrenner et al., 2011; Humayun et al., 2012; Ayton et al., 2014; Chuang et al., 2014; Shivdasani et al., 2014; Goetz and Palanker, 2016). Blindness results from large-scale degeneration of photoreceptors in the outermost portion of the retina. However, a substantial number of inner retinal neurons remain intact (Santos et al., 1997; Medeiros and Curcio, 2001; Mazzoni et al., 2008), thereby providing a target for electric stimulation from the implant. The activation of surviving retinal neurons leads to transmission of a neural signal to the visual cortex which results in a visual sensation. Clinical tests with existing implants have produced functional vision, e.g., some users can recognize objects and/or letters and some report increased mobility as well (Ahuja et al., 2011; Humayun et al., 2012; Dorn et al., 2013; Stingl et al., 2013, 2015). Although these results are encouraging, the overall performance of these devices remains quite limited.

While many factors are likely to contribute to sub-optimal performance, a key limitation is thought to arise from the inability of the implant to create meaningful patterns of neural activity, e.g., physiological-realistic patterns that are recognizable to downstream visual centers. In the healthy retina, each of at least a dozen different types of retinal ganglion cells (RGCs, retinal output neurons) extract different features of the visual world and use distinct patterns of spiking to convey information to higher visual centers (Baden et al., 2016). For example, ON-Sustained RGCs elicit a burst of spikes that persists for the duration of a bright stimulus while OFF-Transient RGCs remain quiet in response to the same stimulus but spike briefly when the stimulus is turned off. It has proven challenging to re-create this diversity in spiking with electric stimulation and the transmission of non-physiological signals to the brain is likely to be difficult to interpret. Several recent studies suggest however, that novel stimulation strategies may be useful for replicating one or more key elements of physiological signaling, e.g., low-frequency sinusoidal stimulation re-creates the out-of-phase spiking that occurs naturally between ON and OFF RGCs (Freeman et al., 2010; Twyford and Fried, 2016). Other approaches to improve the match to physiological signaling have been reported (Cai et al., 2013; Jepson et al., 2014a,b; Twyford et al., 2014; Lorach et al., 2015; Twyford and Fried, 2016; Ho et al., 2018) with the hope that the re-creation of more “natural” signaling patterns in RGCs will lead to more natural responses in downstream visual centers and ultimately to better clinical outcomes.

Unfortunately, it is not yet possible to produce many of these specialized waveforms with current-generation implants and so the clinical effectiveness of these new strategies remains largely unexplored. The neural response arising in visual cortex is thought to be better correlated to perception (than the neural activity in the retina) (Salzman et al., 1990; Knierim and van Essen, 1992) and so here, as a first step toward determining the efficacy of these new stimulation strategies, we explore the responses of cortical neurons to retinal stimulation. Previous studies that have examined cortical responses arising from electric stimulation of the retina have largely focused on the spatial extent of activation from single channel stimulation, or, on the spatial interactions arising from simultaneous stimulation of two or more neighboring electrodes (Wilms et al., 2003; Sachs et al., 2005; Walter et al., 2005; Eckhorn et al., 2006; Wong et al., 2009; Cicione et al., 2012; Sun et al., 2018). Further, much of this previous work has incorporated measurements of electrically evoked potentials (EEPs) or local field potentials (LFPs), i.e., measurements that reflect population responses from large numbers of cells instead of direct measurements from single cells. Surprisingly, little is known about how neurons that comprise each of the six layers of visual cortex respond to stimulation. Because local computations are transmitted from neurons of layers 4, 2/3, 5, and 6, (Douglas and Martin, 2004), it seems particularly important to understand how the neurons in each of these layers are shaped by the parameters of (retinal) stimulation. It is also important to understand whether stimulation strategies that reproduce key elements of physiological signaling in the retina, e.g., selective activation of ON vs. OFF RGCs (Freeman et al., 2010; Cai et al., 2013; Twyford et al., 2014; Twyford and Fried, 2016) result in better matches to natural signaling in the cortex, e.g., out-of-phase firing in ON vs. OFF cells. This is especially intriguing because better matches to the physiological signaling in cortex could be associated with improved clinical outcomes.

Here, we measured the single unit activity arising in neurons of each layer of mouse visual cortex in response to electrical stimulation of the retina. A 16-channel penetrating microprobe allowed simultaneous recordings from multiple layers of the visual cortex; layers were identified by the depth of penetration as well as by the level of spontaneous activity. Cells were further classified as excitatory or inhibitory according to the shape of their waveform and into ON, OFF, or ON-OFF cells according to the polarity of their response to light. After classification, we investigated responses of individual types as a function of the parameters of stimulation (pulse amplitude and pulse rate). We also explored the efficacy with which physiological-like patterns in the retina are transmitted to cortex.

## Materials and Methods

### Animal Preparation

Experiments were performed on adult (age 2–6 months) male C57BL/6 mice (The Jackson Laboratory, United States). This study was carried out in accordance with the recommendations of all federal and institutional guidelines. The protocol was approved by the Institutional Animal Care and Use Committee of the Massachusetts General Hospital (MGH). The mice were housed in the animal facility of MGH under a 12-h light/dark cycle. Each mouse was anesthetized by an intraperitoneal injection of a mixture of Ketamine (100 mg/kg, Henry Schein Animal Health, United States) and Xylazine (10 mg/kg, Akorn Inc., United States). Body temperature was maintained at 37.5°C by a heating pad. The depth of anesthesia was evaluated every 30–60 min by testing the paw withdrawal reflex, the eyelid reflex and whisker movements; Ketamine (100 mg/kg, ∼50% of the initial Ketamine-Xylazine dose) was redosed as needed. After completion of all subsequent testing, the mouse was removed from the stereotaxic frame and euthanized via cervical dislocation.

### *In vivo* Electrophysiological Recording

**FIGURE 1:**
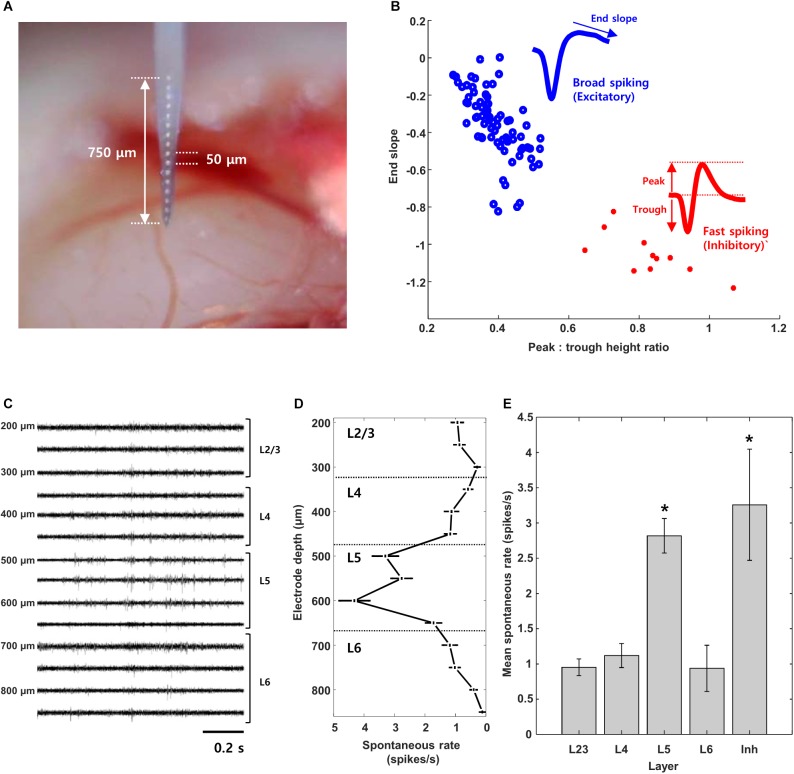
Layer and cell type classification from multisite recording of mouse visual cortex. **(A)** Photograph of the 16-channel linear multisite probe used for insertion into and recording from mouse visual cortex. **(B)** Scatter plot of spike waveform features used to classify cells into excitatory (broad spiking, *n* = 74) and inhibitory (narrow spiking, *n* = 11) types; the average waveforms of broad and fast spiking cells are shown as well. **(C,D)** Raw waveforms and mean spontaneous rate recorded simultaneously from 14/16 channels during a single insertion of the probe. **(E)** Mean spontaneous rate of excitatory cells from each layer and of inhibitory cells pooled across all layers. (L2/3: 15 cells, L4: 18 cells, L5: 29 cells, L6: 16 cells, Inh.: 12 cells), Error bars denote standard error mean (SEM). ^∗^indicates *p* < 0.05.

After the mouse was anesthetized, the animal was moved to the recording setup in a darkened room and placed on a stereotaxic frame (SR-9M-HT, Narishige, Japan). Ear bars were positioned into the auditory canals and the scalp was retracted for a craniotomy over primary visual cortex (2-mm diameter); the dura mater within the exposed area was carefully perforated with a thin needle (30 G) and a forceps. Because stimulation was always presented to the right eye (see below), the craniotomy was performed in the left cortical hemisphere. The exposed cortex was rinsed with PBS to clear any residual debris before insertion of the recording electrode. Recordings were made with a 16-channel silicon microprobe (a1x16-3mm50-177, NeuroNexus Technologies, United States); individual electrodes on the microprobe were 15 μm in diameter with 50 μm center-to-center spacing. In some experiments, a single tungsten microelectrode was inserted instead (WE30012.0F3, Microprobes for Life Science, United States). Recording electrodes were oriented orthogonally to the cortical surface and lowered using a micromanipulator (SMM-100, Narshige, Japan) (Figure 1A). The position of each electrode within the visual cortex was estimated from the depth readout of the micromanipulator as well as by checking the position of the uppermost electrode and its distance from the cortical surface. The depth of individual cortical layers was based on Olsen et al. (2012) and defined as (in μm): L2/3, 100–350; L4, 350–450; L5, 450–650; and L6 >650. Final calibration of electrode depth was made from the rate of spontaneous firing as measured on individual electrodes (see Figure 1): L5 is known to have the highest rate of spontaneous firing (Niell and Stryker, 2008). The recording array typically spanned the full depth of the visual cortex. After the electrode was inserted, the area was covered with 2.5% agarose or PBS to prevent drying and the electrode was allowed to “settle”for 30–45 min before recordings were started. Electrode signals were recorded using an amplifier (Model 3500, A-M Systems, United States) and a data acquisition system (Micro 1401-3, CED, United Kingdom) with software (Spike2, CED, United Kingdom). The extracellular signal was filtered from 100 to 10 kHz and sampled at 25 kHz. All signals were stored on a hard drive and analyzed off-line with custom software written in MATLAB (MathWorks, United States).

### Visual and Electrical Stimulation

Visual stimuli consisted of full-field flashes that were generated and controlled by custom software written in LabView (National Instruments, United States) and MATLAB (MathWorks, United States). Each stimulus was delivered at least 30 times (referred to as “repeats”); peristimulus time histograms (PSTHs) were then generated to facilitate the analysis of responses and the classification of cell types. The visual stimulus was displayed on a monitor (Hewlett Packard, HP ZR22w, refresh rate 60 Hz) placed 25 cm from the mouse with a viewing angle of 45° from the center of the monitor (toward the right eye of the mouse).

Electrical stimulation was delivered extraocularly via a platinum-iridium wire (model: 78000, A-M systems, United States); the diameter of the wire was 127 μm, giving an estimated surface area of 12,667 μm^2^. The wire was positioned on the surface of the cornea of the right eye using a micromanipulator (SM-25A, Narishige, Japan). The return wire was inserted under the skin behind the neck. Pulse stimuli were generated by a STG8002 stimulator (Multichannel Systems GmbH, Germany) and MC_Stimulus software (Multichannel Systems GmbH, Germany). Stimulus pulses were cathodal-first, biphasic, charge balanced pulses that were rectangular in shape with no interval between the cathodal and anodal phases. Pulse duration was fixed at 2 ms unless otherwise specified. Pulse rate ranged from 1 to 5 pulses per second (PPS) and pulse amplitudes ranged from 20 to 300 μA. For sinusoidal stimulation, frequency ranged from 1 to 5 Hz; the amplitude of all sinusoids was fixed to 100 μA and 20 cycles were delivered regardless of stimulation frequency.

### *In vitro* Electrophysiological Recording

For *in vitro* patch clamp recording, wild type (C57BL/6J) mice were anesthetized with isoflurane and subsequently euthanized by cervical dislocation. Eyeballs were harvested, retinas were dissected from the eyecup and mounted, photoreceptor side down, onto a recording chamber. The retina was subsequently perfused with oxygenated Ames medium (Sigma-Aldrich, United States) at a flow rate of 2–3 ml/min for the duration of the experiment. Temperature was kept at ∼34°C. Small holes were made in the inner limiting membrane in order to obtain access to RGC somata. Spiking responses were obtained using loose (cell-attached) patch recordings. Patch electrode resistance was ∼6–8 MΩ. The visual stimulation was projected from below onto the photoreceptor outer segments using an LCD projector (InFocus, United States). Visual stimulation consisted of bright spots on neutral (gray) background with diameters ranging from 100–1500 μm and presented for 1 s. ON and OFF-α S cells were targeted by their large somata (diameter >15 μm) and identified by their strong sustained light responses. Stimulus control and data acquisition were performed with custom software written in LabView (National Instruments, United States) and Matlab (Mathworks, United States). The electrical stimulation delivered via a 10 kΩ platinum–iridium electrode (MicroProbes, United States); the exposed area at the electrode tip (no Parylene-C insulation) was conical with an approximate height of 125 μm and base diameter of 30 μm, giving a surface area of ∼5,900 μm^2^. Stimulating electrodes were positioned 30 μm above the inner limiting membrane; the tip of the electrode was raised by micromanipulator after touching the surface of the inner limiting membrane. Two silver chloride-coated silver wires served as the return; each was positioned ∼8 mm from the targeted cell and ∼6 mm from the other wire. The electric stimuli were applied by a stimulus generator (STG 2004, Multi-Channel Systems MCS GmbH, Germany). For biphasic stimulation, stimulation parameters such as pulse duration (2 ms per phase) and pulse rate (1–5 Hz) were same with those used for *in vivo* experiment. Stimulation amplitude was fixed at 100 μA. For the electrical sinusoidal stimulation, stimulation frequency from 1 to 5 Hz was used. Pulse and sinusoidal stimuli were controlled by Multi-Channel Systems STG2004 stimulator (Multichannel Systems GmbH, Germany) and MC_Stimulus software (Multichannel Systems GmbH, Germany). Data were recorded using an Axopatch 200B amplifier (Molecular Devices, United States) and digitized by a data acquisition card (PCI-MIO-16E-4, National Instruments, United States). The timing of individual spikes was detected as the depolarization (negative) peak of each spike in the raw trace.

### Data Analysis

Spikes were detected by applying a negative threshold to the recorded signal; the timing of individual spikes corresponded to the most negative point of the waveform and therefore, the latency values reported here are ∼0.5 ms slower than actual onsets. Activity from multiple cells was often captured on a single electrode during the experiment and principal component analysis (PCA) was used for spike sorting (custom software written in MATLAB). Cells were used only if their spike waveforms could be unequivocally separated from other cells on the same electrode. After spike sorting, the average spike waveform was determined for each cell and saved as a template; this allowed verification that the same cell was being consistently recorded throughout an experiment. For example, the average waveform from the spontaneous activity portion of an experiment could be compared to the waveforms from visual stimulation as well as to the responses to electric stimulation. Cells were classified as ON, OFF, or ON-OFF based on their responses to full-field light stimuli.

Features of the extracellular spike waveform were used to distinguish excitatory and inhibitory neurons (see Figure 1B) and were based on previous work (Csicsvari et al., 1999; Bruno and Simons, 2002; Andermann et al., 2004; Bartho et al., 2004; Niell and Stryker, 2008; Rummell et al., 2016). In particular, we found consistently good separation using (i) the height of the positive peak relative to the initial negative trough, and, (ii) the slope of the waveform from the first peak to the baseline (Niell and Stryker, 2008). Amplitudes of each average spike waveform were normalized before classification.

The electric artifact was divided into two periods (Figure 3A, labeled as periods “i” and “ii”). During the first period which is the actual duration of the stimulation pulse, the amplifiers were saturated and spikes could not be detected. After the first period, the artifact was still prevalent but spikes could nevertheless be observed. The use of an amplitude threshold was not effective for extracting individual spikes and so we utilized a traditional artifact removal technique (Hashimoto et al., 2002) in which a template of the average stimulation artifact waveform is subtracted from the raw waveform in individual traces. Since the artifact size and shape could vary slightly across trials, some residual artifact often remained after subtraction and could result in false positives. As a result, the time period from 0–7 ms after the pulse onset was “flattened” by zero-padding [indicated by (ii) in Figure 3A]. Spikes could reliably be detected after 7 ms and their waveforms visually compared to the template waveforms recorded during spontaneous activity and/or visual stimulation (Spike waveforms shown in Figure 3B–E).

To quantify the strength of a given electrically elicited response, the number of spikes elicited within 100 ms of the pulse onset was counted and averaged across all repeats of a given stimulus. For statistical analysis, the Student (independent sample) *t*-test was used, *p* < 0.05 was considered as significant (^∗^). In figures presenting the median of data, error bars denote the standard error of the mean.

During patch recordings (the retinal *in vitro* experiments), we record in voltage-clamp mode and so we capture currents (not voltages); with this approach, the polarity of the raw stimulus artifact appears as a negative deflection for anodal stimuli and a positive deflection for cathodal stimuli. This is inverted from typical convention; previous studies that have performed similar experiments (Freeman et al., 2010; Twyford and Fried, 2016), have made note of the anomaly. In the cortical (*in vivo*) experiments, the polarity of the stimulus artifact is not inverted and so the raw waveforms from the two sets of experiments would appear opposite to one another. We felt that this would be confusing in Figure 8, where both *in vitro* (Panels A–D) and *in vivo* (Panels E–H) are presented together, i.e., if positive waveforms indicated cathodal stimuli in one part of the figure and negative stimuli in another, it would make the results of the figure difficult to interpret. Therefore, we artificially “inverted” the retinal *in vitro* stimulus waveforms. This was done by low-pass filtering the raw waveform to extract the stimulus waveform, inverting it, and then adding it to a high-pass filtered version of the raw waveform (to capture the spiking responses without the stimulus artifact). While this depicts the same polarity for cathodal and anodal stimuli in both sets of experiments, it has the adverse effect of making the *in vitro* retinal results appear different from earlier work (Freeman et al., 2010; Twyford and Fried, 2016).

## Results

A 16-channel implantable microprobe was used to obtain simultaneous recordings of single unit activity from multiple layers of mouse visual cortex. The results below are based on *in vivo* recordings from 126 cortical neurons (L2/3: 24, L4: 32, L5: 37, L6: 33) obtained from 33 adult mice and *in vitro* recordings from 17 RGCs obtained from 5 additional mice.

### Layer and Cell Type Classification

Similar to a previous study (Niell and Stryker, 2008), we used the end slope and the peak-to-trough height of measured spikes to classify cells as excitatory vs. inhibitory (Methods, Figure 1B); these two parameters resulted in linearly good separability between the two cell types (Andermann et al., 2004; Niell and Stryker, 2008). Similar to previous approaches, cells with broad-spiking waveforms (blue) were classified as excitatory while those with fast-spiking waveforms (red) were classified as inhibitory (Csicsvari et al., 1999; Bruno and Simons, 2002; Andermann et al., 2004; Bartho et al., 2004; Niell and Stryker, 2008; Rummell et al., 2016). Most of the cells we found were excitatory (*n* = 113/126, L2/3: 23/24, L4: 29/32, L5: 33/37, L6: 28/33).

Figure 1C shows a portion of the raw spontaneous activity recorded from 14 channels during a typical experiment. The mean firing rate was calculated for each cell (Methods) and then the average rate of firing across all cells at a given insertion depth was plotted (Figure 1D). Insertion depths of 100–350 μm corresponded to layer 2/3 (L2/3) while depths of 350–450, 450–650 and >650 corresponded to layers 4, 5, and 6 (L4, L5, and L6, respectively) (Olsen et al., 2012). The mean rate of activity for cells at depths of 500–650 μm (L5) was higher than that of layers 2/3, 4, or 6 (*p* < 0.01 for all individual comparisons); the higher level of spontaneous activity observed here (L5) is consistent with previous work (Niell and Stryker, 2008). As the number of inhibitory cells found here was limited (*n* = 13), the mean firing rate was pooled from cells across all layers; the mean inhibitory rate was comparable to that from L5 excitatory neurons and significantly different from the mean rate of other layers (*p* < 0.05 for all individual comparisons) (Niell and Stryker, 2008).

**FIGURE 2:**
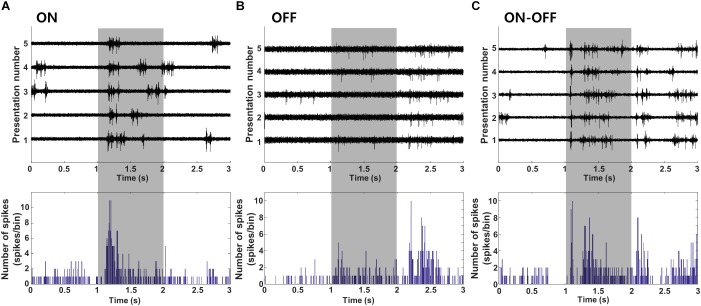
Cell type classification based on visual response. **(A–C)** Cell were classified into ON, OFF, or ON-OFF types based on the polarity of their response to a full-field light stimulus (duration of 1 s indicated by the shaded box). Light stimulation was applied 30 times; raw waveforms for 5 consecutive presentations (out of 30) are shown for a typical cell of each type. PSTHs for all cell types are shown at bottom (time bin = 10 ms).

Most of the V1 neurons we tested showed reliable responses to full field light stimulation (Figure 2). Cells responded to either the onset (Figure 2A, “ON,” *n* = 23, L2/3:7, L4:12, L5: 3, L6:1), the offset (Figure 2B, “OFF,” *n* = 13, L2/3:5, L4:3, L5:3, L6:2) or both the onset and offset of the stimulus (Figure 2C, “ON-OFF,” *n* = 90, L2/3:12, L4:17, L5:31, L6:30). Consistent with earlier studies (Weng et al., 2005; Mace et al., 2015; Bae et al., 2018), cells from this third group exhibited considerable variability in the relative proportion of their ON vs. OFF responses. Table 1 summarizes the complete classification of all cells captured in this study.

**Table 1:** Classification of all cells captured in this study.

		L2/3	L4	L5	L6
ON	Excitatory	6	12	3	1
	Inhibitory	1	–	–	–
OFF	Excitatory	5	2	1	2
	Inhibitory	–	1	2	–
ON-OFF	Excitatory	12	15	29	25
	Inhibitory	–	2	2	5


### Electric Stimulation of the Retina Induces Robust Responses in V1 Neurons

**FIGURE 3:**
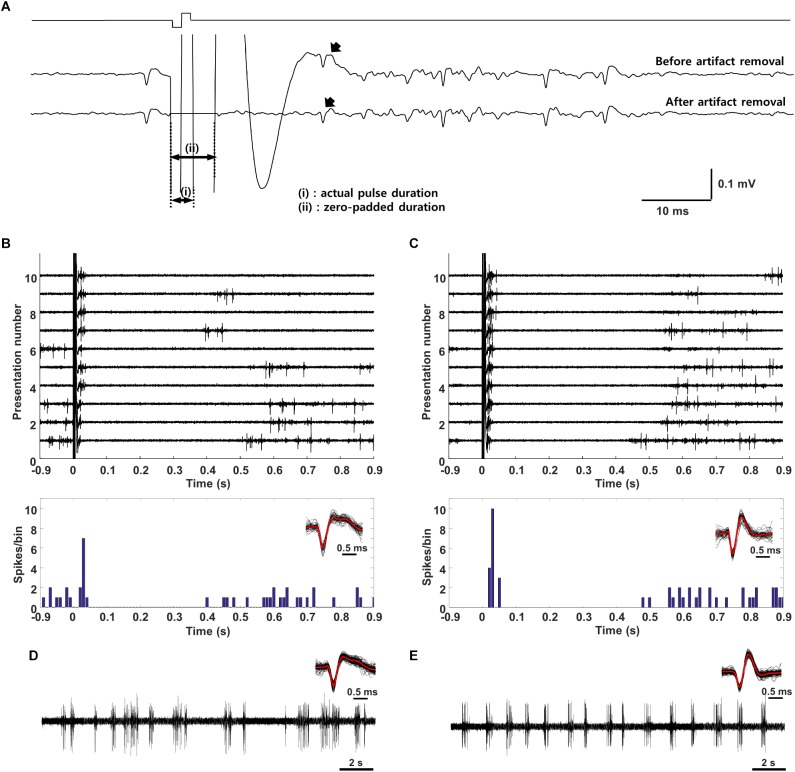
Electric stimulation elicits similar responses in excitatory and inhibitory neurons. **(A)** The stimulus (charge-balanced biphasic pulse, cathodal first, 2 ms/phase, 300 μA) produced an electrical artifact that typically persisted longer than the duration of the stimulus itself. Spikes were sometimes visible within the artifact (top trace, arrow) but were difficult to detect via thresholding (see text); subtraction of the mean artifact from the raw waveform facilitated extraction of such spikes (bottom trace, arrow) but also could result in intermittent false positives during the first 7 ms. To avoid detecting false positives as spikes, this time period was zeroed (bottom trace, period indicated by “ii”). The period labeled as “i” indicates the actual pulse duration. **(B,C)** Raw waveforms (top) and peristimulus time histogram (PSTH) (bottom) of a typical **(B)** L5 excitatory and **(C)** L5 inhibitory neuron. Detected waveforms and their average (red) were overlaid in the inset. (Bottom) **(D,E)** Raw waveforms (spontaneous activity) from the cells in panel **B** and **C**, respectively. The overlay of extracted spikes and the average waveform (red trace) facilitated comparison to electrically and/or visually evoked spikes.

Once cells were classified into layer and type, electric stimulation was delivered to the outer surface of the eye (extraocular stimulation, Methods) and the responses of individual V1 neurons were measured (Figure 3A). The stimulus was a biphasic waveform, 2 ms/phase and cathodal first; these type of stimuli have been shown previously to strongly activate RGCs (Jensen and Rizzo, 2008; Lee et al., 2013). Delivery of the stimulus typically saturated the amplifiers for 4–5 ms and the persistence of an electrical artifact hindered our ability to reliably detect spikes for an additional 2–3 ms following the emergence from saturation (Figure 3A). Although we could reduce the size of the artifact somewhat by digitally subtracting the mean artifact, averaged over many trials, from each raw recording, the residual artifact often led to false positives. To eliminate the possibility of erroneous spikes, we “zeroed out” the response for the first 7 ms following stimulus onset (Figure 3A, bottom trace). After this period, spikes that arose within the remaining portion of the artifact could be reliably captured by our spike detection algorithms (Methods). Spike waveforms detected in response to electric stimulation (Figure 3B, overlay at bottom right) were compared to the waveforms from spikes arising spontaneously (Figure 3D, overlay at top right) or in response to light (not shown); consistency in waveform shape was used to confirm that electrically elicited spikes were indeed from the same cell, e.g., the small movements of the brain those that occurred routinely during experiments did not result in a shift of the recordings to a different cell.

Responses to electric stimulation typically consisted of a brief burst of spiking that occurred within the first 50 ms following stimulus onset and was followed by a 400–500 ms period during which there was little or no spiking (Figure 3B). The elimination of spontaneous activity suggests the presence of a strong inhibitory signal during this period and is consistent with much previous work showing a slow-acting but strong wave of inhibition triggered by electrical and other forms of artificial stimulation (Logothetis et al., 2010). The end of the quiet period was marked by the recovery of spontaneous activity. In a few cells with very low spontaneous rates, it could be difficult to accurately determine the duration of the quiet period (not shown). Responses to electric stimulation were similar in both excitatory and inhibitory cells, e.g., compare the responses in Figure 3B,C. The timing of the initial burst of spikes in cortical neurons is consistent with the brief, short-latency burst of spikes arising in many different types of RGCs [(Im and Fried, 2015; Werginz et al., 2018), see also Figure 5].

**FIGURE 4:**
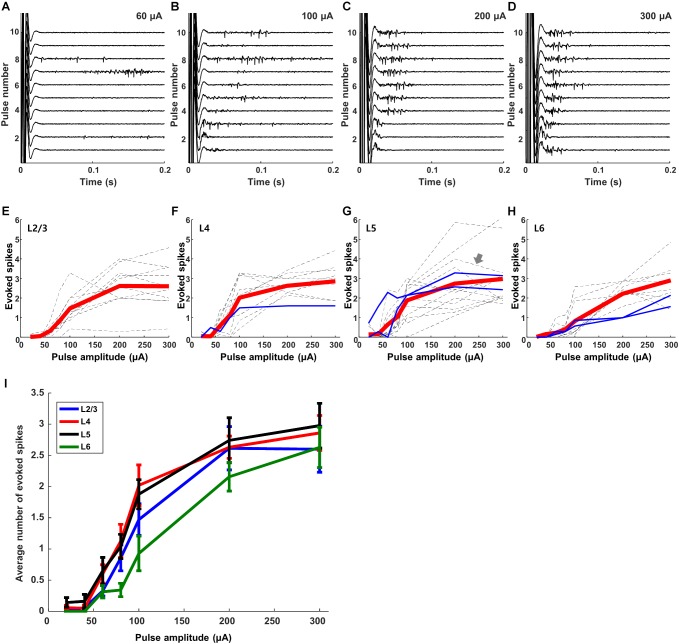
Responses to electric stimulation are sensitive to pulse amplitude. **(A–D)** Raw waveforms recorded in response to electric stimulation from a typical L5 excitatory cell. Each panel shows the response to 10 consecutive pulses; amplitudes are 60, 100, 200, and 300 μA in the 4 panels, respectively. **(E–H)** Dotted gray and blue lines indicate average responses as a function of amplitude from individual excitatory and inhibitory cells, respectively (L2/3: 10 cells, L4: 10 cells, L5: 14 cells, L6: 10 cells). Thick red lines in each graph indicate the average response across all cells within the layer. **(I)** Overlay of the average responses from each layer (the red lines from panels **E–H**).

Responses to electric stimulation were sensitive to the strength of the stimulus pulse. Figure 4 shows the responses from a typical L5 excitatory neuron for amplitudes ranging from 60–300 μA. There was some variability across trials, especially for weaker stimuli, e.g., compare responses across the 10 repeats of each stimulus level in panels A–D, but we did not attempt to identify the source of variability. We plotted the average number of spikes elicited within the first 100 ms of each trial (Methods) as a function of stimulus amplitude for each individual cell (dashed lines in Figure 4E–H; the arrow in Figure 4G indicates the cell of panels A–D). Mean responses were generally similar for the cells of a given layer (the average response across all cells in the layer is shown in red.) Overlay of the average responses from all layers (Figure 4I) reveals that the sensitivity to amplitude was also similar across layers, including a similar peak level of peak response. The few inhibitory cells that were tested in this experiment (blue lines), exhibited responses that were consistent to those of excitatory cells. We did not observe non-monotonic responses, i.e., responses that increased to the initial increase in stimulus amplitude but then decreased for further increases in amplitude (Barriga-Rivera et al., 2017) (Discussion).

### Electrically Elicited Responses Are Suppressed With Increasing Pulse Rate

**FIGURE 5:**
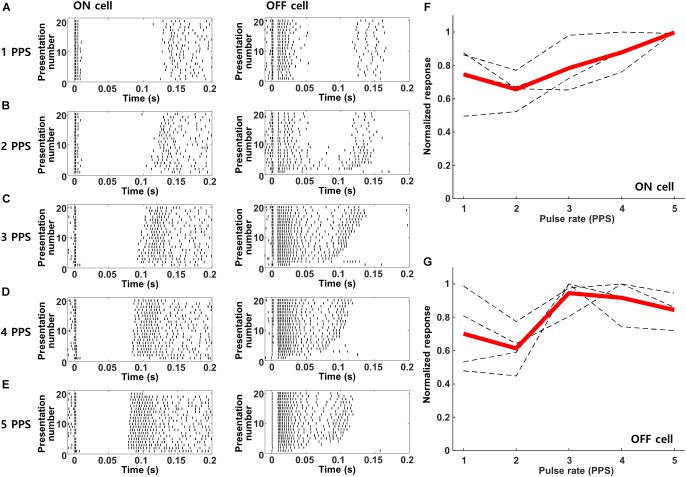
Retinal ganglion cells maintain robust responses to electric stimulation from 1–5 PPS. **(A–E)** Raster plots of a typical ON (left) and OFF (right) RGC in response to pulse rates of 1–5 PPS (biphasic pulse, cathodal first, 2 ms/phase, 100 μA). **(F,G)** Normalized response strength of 4 ON and 4 OFF RGCs, respectively. Dotted gray lines are the response curves from individual RGCs average over 20 repeats; thick red lines indicate the average response of each cell type.

The prolonged period of inhibition followed each response to electric stimulation (Figure 3B,C) suggests that responses to repetitive stimulation may be diminished, at least for rates in which the new stimulus pulse overlaps with the inhibitory signal from the previous pulse. Prior to evaluating the responses of cortical neurons however, we first tested the response of mouse RGCs to increasing rates of stimulation so as to establish the baseline signal leaving the retina. Figure 5 shows the responses from ON and OFF RGCs in the mouse retinal explant (Methods) to stimulation rates ranging from 1–5 PPS; waveforms were biphasic, cathodal first, and 2 ms/phase). Similar to previous studies (Lee et al., 2013; Im and Fried, 2015; Werginz et al., 2018), responses to these types of relatively long-duration stimuli elicited multiple bursts of spiking that were fairly consistent from trial to trial. As described previously, the onset latencies for bursts were different in ON vs. OFF RGCs (compare burst timing in the left vs. right panels). Individual bursts remained robust and largely consistent as the rate of stimulation was increased from 1 to 5 PPS, although there were some variations in the timing as well as the duration of individual bursts. We counted the number of spikes elicited within the first 200 ms following each stimulus and averaged the results across all ON and OFF cells (Figure 5F,G, respectively). The results indicated small variations in the strength of the response across this range of frequencies but there was no systematic reduction in responsiveness with increasing rate, at least up to 5 PPS.

**FIGURE 6:**
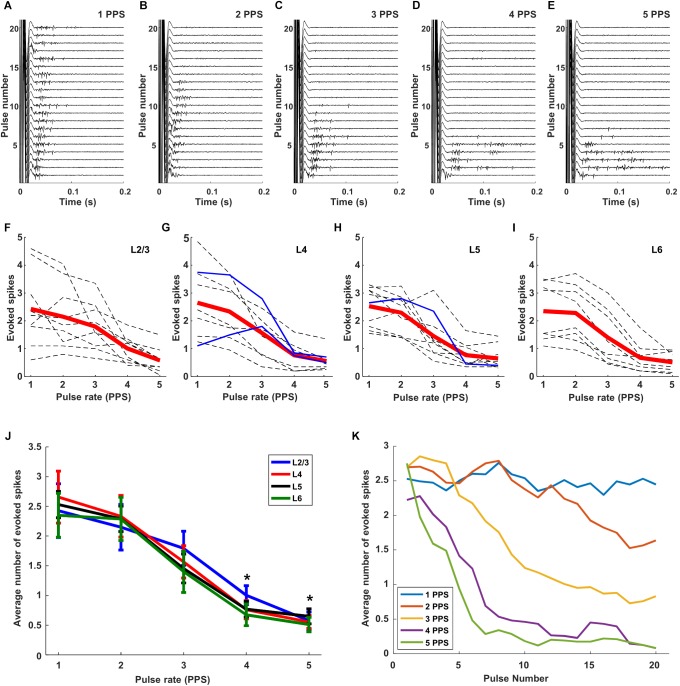
Responses are suppressed by increasing rates of stimulation. **(A–E)** Raw responses from a typical L5 excitatory cell to 20 consecutive pulses at 300 μA; rates of 1, 2, 3, 4, and 5 PPS, respectively, were applied. **(F–I)**. Dotted gray and blue lines are the mean responses from individual excitatory and inhibitory cells, respectively (L2/3: 9 cells, L4: 9 cells, L5: 10 cells, L6: 8 cells). Thick red lines indicate the average response across all cells from the layer. **(J)** Overlay of average response curves from all layers. ^∗^indicates *p* < 0.05. **(K)** Response strength versus stimulus number averaged across all layers. Error bars omitted for clarity.

When we delivered the same stimulus extraocularly at 1 PPS and measured the responses of cortical neurons *in vivo*, responses were observed for all 20 pulses (Figure 6). The amplitude of stimulation was fixed to 300 μA so as to ensure a strong uniform response across all layers (Figure 4I). Similar to the RGC responses, there was some variability across trials in both response strength as well as the timing of individual spikes (Figure 6A). When the stimulus rate was increased to 2 PPS however, responses were elicited by the first 13 pulses but not for the subsequent 7 (Figure 6B). At even faster rates of stimulation, the number of trials that elicited responses continued to decrease: spikes were elicited for only the first 10 pulses at a rate of 3 PPS (Figure 6C) and for only 6 and 7 pulses at rates of 4 and 5 PPS, respectively (Figure 6D,E). It is interesting to note that the reduced sensitivity to rates of 3, 4, and 5 PPS arose despite the fact that retinal response levels were not similarly reduced at these same rates (Figure 5C–E). Thus the loss of sensitivity in cortical neurons is not due to a corresponding reduction in output signal from the retina, but instead suggests that the loss arises during transmission of the retinal signal to the cortex. Our findings do not reveal the source of this loss in sensitivity, but it is interesting to note that responses in L4 neurons were similar to those from other layers (not shown but see Figure 6J), suggesting that the loss occurs prior to arrival at the cortex. It will be interesting in future experiments to record from visual neurons that receive input directly from RGCs (e.g., at the superior colliculus or lateral geniculate nucleus) so as to examine their sensitivity to the rate of stimulation; this may help to pinpoint the location at which the sensitivity to higher rates of stimulation is lost. Similar types of suppressive effects have been described in the retina (referred to as desensitization) although they occur at higher rates of stimulation (Jensen and Rizzo, 2007; Freeman and Fried, 2011). Retinal desensitization is thought to be triggered by the strong inhibitory signals that persist longer than the intervals between consecutive stimuli and it is likely that the prolonged inhibitory signal observed in cortical neurons in earlier experiments (Figure 3) is similarly tied to the loss of responsiveness seen here. Whereas retinal inhibitory periods persist for <100 ms (Fried et al., 2006), the inhibitory signals seen earlier in cortical neurons (Figure 3) persist for several hundred milliseconds and is consistent with the fall-off of sensitivity at lower-frequencies. To quantify the level of desensitization observed here, we determined the average number of electrically elicited spikes across all 20 repeats for each rate of stimulation. Each dotted line in Figure 6F represents the average response from an individual cell in Layer 2/3; the responses from cells in layers 4, 5, and 6 are shown in Figure 6G–I, respectively. The red line is the mean response across all cells in the layer. There was a monotonic decrease as stimulus rate increased from 1 to 5 PPS although, once again, there was significant variability across cells. Only three inhibitory cells (two cells in layer 4 and one in layer 5) were tested in this experiment (solid blue lines), their responses were generally similar to those of excitatory neurons.

We overlaid the average plot of response strength vs. pulse rate from each layer (Figure 6J) and found that both the overall strength of the response as well as the sensitivity to pulse rate were quite similar across layers. When the mean levels were statistically compared across layers, the responses to 5 PPS stimulation were found to be significantly lower than those to 1 PPS (*p* < 0.005) for all layers. Response strengths at 4 PPS were also significantly lower than those at 1 PPS (*p* < 0.05 for layer 4, *p* < 0.005 for layer 2/3, 5, 6). There was no significant difference in response strength for rates of 1 vs. 2 PPS. A comparison of the response strength as a function of pulse number provided further confirmation that there was minimal desensitization for rates of 1 PPS (Figure 6K); as the rate of stimulation increased however, the onset of desensitization occurred earlier, and its effect was stronger. At the fastest rates tested here (5 PPS), desensitization was already evident in the response to the second pulse and responses were almost completely suppressed by the 5th pulse.

**FIGURE 7:**
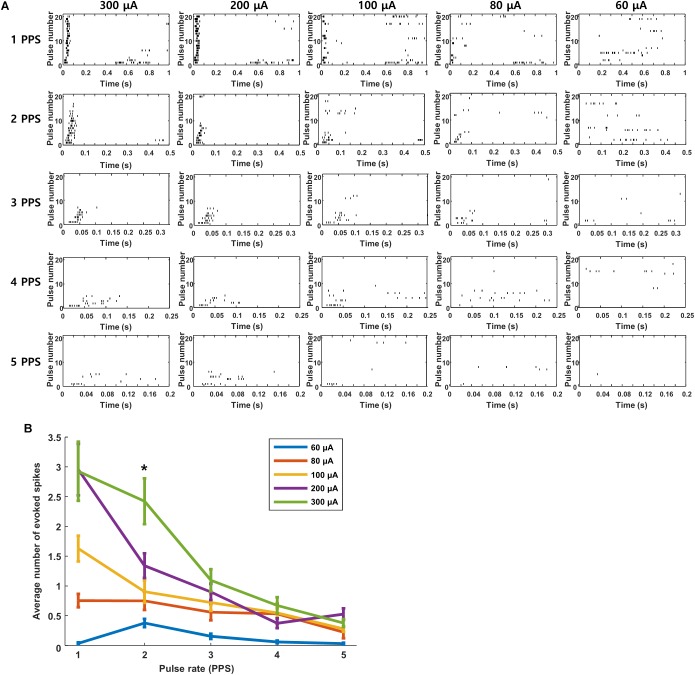
Desensitization is not caused by high stimulus strength. **(A)** Raster responses to electrical stimulation at pulse rates of 1–5 PPS and amplitudes of 60–300 μA in a typical cell. **(B)** Average response strength across all cells as a function of pulse rate; each data point is the average of 11 cells across all layers (L2/3: 2 cells, L4: 2 cells, L5: 4 cells, L6: 3 cells.). ^∗^indicates *p* < 0.05.

We questioned whether the high levels of desensitization seen in these experiments might be arising from the relatively strong stimuli that were used to ensure robust responses from the extraocular stimulus. If so, we reasoned that lower amplitudes might reduce the level of desensitization. Each plot in Figure 7A is a raster response to 20 identical stimuli; the rate of stimulation is fixed for each row of plots while the amplitude decreases across columns (from left to right). Consistent with the results of Figure 6, responses were consistently robust for a stimulus rate of 1 PPS and a pulse amplitude of 300 μA but they became weaker and less consistent when stimulus strength was reduced to 200 μA and even weaker still at 100 μA. Even with the weaker responses however, there was still a clear reduction in both strength and consistency as the rate of stimulation increased. The persistence of desensitization at weaker stimulus amplitudes suggests that the reductions observed in Figure 6 were not due to the use of a strong stimulus. At even weaker stimulus levels, e.g., 80 μA, responses were barely detectable, even at 1 PPS, and so it was difficult to assess whether desensitization was present. The response patterns seen in Figure 7 were highly similar to those from cells in other layers (not shown). Averaging responses across all layers (Figure 7B) confirmed that desensitization was strongest for strong stimulus levels but could be observed for any level of stimulation that was strong enough to induce a response. We conclude therefore that the desensitization observed here is not mediated solely by the strength of the stimulus.

### Low-Frequency Sinusoids Elicit Out-of-Phase Responses in ON vs. OFF Cells

**FIGURE 8:**
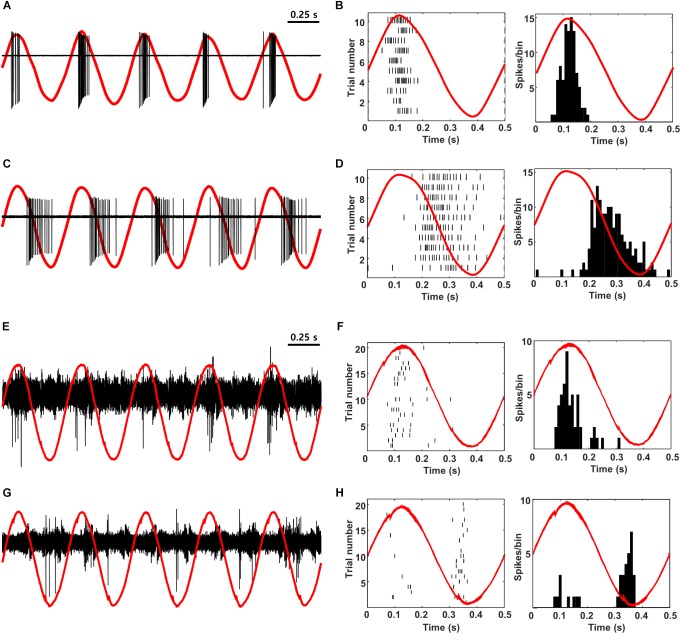
ON vs. OFF selectivity is preserved during propagation from the retina to V1. **(A,C)** Raw *in vitro* response (black) and stimulus waveform (red) from a typical ON and OFF RGC in response to 2-Hz sinusoidal stimulation. **(B,D)** Raster plot (left) and PSTH (right) for the same cells. The red traces are a single period of the stimulus waveform aligned to showing timing of the responses. **(E,G)** Raw response (black) and stimulus waveform (red) from a typical L4 ON cell **(E)** and a typical L2/3 OFF cell **(G)** in response to 2-Hz sinusoidal stimulation (100 μA). **(F,H)** Raster plot (left) and PSTH (right) for the same cells. The red traces are a single period of the stimulus waveform aligned to showing timing of the responses. Note that anodal phases appear as upward and cathodal as downward in all panels (Methods).

Low-frequency sinusoidal waveforms activate OFF RGCs during the cathodal phase of the stimulus and ON RGCs during the anodal phase (Freeman et al., 2010; Twyford and Fried, 2016). The ability to reproduce the out-of-phase firing that occurs naturally in RGCs is intriguing and so we questioned whether the out-of-phase firing could be reliably transmitted to cortex, e.g., would ON and OFF cells in cortex exhibit out-of-phase responses? To explore this, we first verified that low-frequency sinusoidal stimulation of mouse retina elicited similar out-of-phase responses to those described previously in rabbit (Figure 8A–D). 2 Hz stimulation from a small Pt-Ir electrode (10 kΩ) that was positioned close to the surface of the retinal explant produced burst spiking in ON cells during the peak of the anodal phase of the stimulus (Figure 8A,B) but little or no spiking in OFF RGCs at the same time (Figure 8C,D). Instead, OFF RGCs responded strongly during the cathodal phase while ON cells were quiet. In subsequent *in vivo* experiments, we delivered electric sinusoidal stimulation at rates of 1–5 Hz extraocularly while measuring the resulting responses in both ON and OFF cells of V1 (Figure 8E–H). Low-frequency sinusoids produced robust spiking in both ON and OFF types of cortical neurons (Figure 8E,G, respectively) but it was difficult to detect any significant differences from direct observation of the raw waveforms. Converting the timing of elicited spikes to the phase of the stimulus however, revealed that the responses in ON cells indeed occurred during the anodal phase of the stimulus while OFF cells responded during the cathodal phase (PSTHs in Figure 8F,H; 13/17 ON cells; 9/9 OFF cells). These recordings suggest that the out-of-phase responses generated artificially in RGCs are indeed faithfully transmitted to higher visual centers. The ability to differentially drive ON vs. OFF channels along the entire visual pathway is intriguing because cortical responses are thought to correlate better to perception and so the ability to selectively target the individual channels offers the hope of higher quality percepts.

## Discussion

Our investigation into the response of visual cortical neurons to electrical stimulation of the retina yielded several important insights. First, we found that responses in all cortical neurons were generally brief, persisting for ∼50 ms, and followed by a prolonged period of inhibition (lasting up to a few hundred milliseconds). The relatively short response period was curious given the longer response durations in upstream RGCs. Second, the responses to biphasic pulse stimulation were highly similar across layers and cell types. Third, responses to electric stimulation were highly sensitive to the rate at which stimulation was delivered, e.g., they were significantly reduced for rates as low as 3 PPS. This loss in sensitivity was not mediated within the retinal circuitry as ganglion cell responses remained consistent for rates up to 5 PPS. Finally, selective targeting of ON vs. OFF RGCs (via novel stimulation strategies) led to selective responses in ON vs. OFF cortical neurons, i.e., signaling properties were preserved during propagation to higher visual centers. It is important to note that both ketamine and xylazine can alter cortical responsivity (Bengtsson and Jorntell, 2007; Ordek et al., 2013) but given that anesthesia may not strongly alter the tuning of neurons in primary visual cortex (Lamme et al., 1998; Schanze et al., 2006; Niell and Stryker, 2010), the responses observed here may be representative of those in the awake, behaving animal. Each of these findings is discussed below.

### Cortical Responses to Electric Stimulation Are Brief

The responses to pulsatile electrical stimulation were brief, typically persisting for ∼50 ms but always less than 100 ms. While the limited duration is consistent with previous studies (Cicione et al., 2012; Shivdasani et al., 2012; Nimmagadda and Weiland, 2018), they were still somewhat surprising given the prolonged duration in retinal neurons, e.g., spiking responses persisted for ∼150 ms in OFF RGCs and almost 200 ms in ON RGCs (Figure 5A). While it is possible that the different durations arise from methodological differences (*in vitro* measurements in the retinal explant utilizing a small stimulating electrode vs. *in vivo* stimulation utilizing a much larger extraocular stimulating electrode), previous studies using a wide range of electrode locations, also report relatively short response durations using smaller, implanted electrodes (Eckhorn et al., 2006; Cicione et al., 2012; Shivdasani et al., 2012; Villalobos et al., 2014). These results suggest that spike bursts with onset latencies >100 ms, e.g., the later bursts in both ON and OFF RGCs (Figure 5), do not effectively drive cortical neurons and thus short-latency spike bursts in RGCs are more relevant to cortical responses and probably to psychophysical percepts. Our results do not reveal the reason for the loss of the later bursts but the prolonged period of suppression following the initial burst of spiking in cortical neurons (Figure 3) raises the possibility that subsequent input to cortical neurons is rendered ineffective by the sustained inhibitory signal. Studies in other CNS neurons have described a similar type of sustained inhibitory signal that arises from artificial stimulation (Logothetis et al., 2010).

Similar to earlier studies using both multi-unit recordings (Cicione et al., 2012; Shivdasani et al., 2012; Barriga-Rivera et al., 2017) and EEPs (Chowdhury et al., 2005, 2008), we found that increasing the amplitude of the stimulus pulse resulted in an increase in the number of spikes generated by cortical neurons. The magnitude of RGC responses have also been shown to be sensitive to stimulation strength (Lee et al., 2013) and so it is likely the stronger cortical responses observed here arise directly from stronger responses in RGCs. Cortical responses peaked at 3–4 spikes for even the strongest biphasic pulses we delivered, a level that is comparable to that from a recent report in cat (Barriga-Rivera et al., 2017) although we did not find evidence of non-monotonic responses in some cells as they observed. The similarities in sensitivity to previous reports, including similar magnitudes of overall response strength suggests that our use of extraocular electrodes for stimulation elicits comparable activation of the retina to that from electrodes implanted in the eye. Because we recorded responses with only a single probe, our study does not reveal response variability across different regions of V1 (Cicione et al., 2012) and it is likely that there would be considerable difference if small stimulating electrodes, close to the retina, were compared to the extraocular stimulation used here; it will be useful to perform follow-up studies that incorporate such electrodes in a blind animal model. Testing a chronic implant in an awake behaving animal is also desirable as it will eliminate the potential for response alteration due to anesthesia.

### Responses to Electric Stimulation Are Similar Across Cell Types and Layers of V1

We found here that the cortical responses to electric stimulation of the retina were highly consistent across all types of neurons and all layers of the visual cortex (Figure 4, 6). Prior to evaluating the responses to electric stimulation, we first classified cortical neurons into previously established classes. This included (1) the use of visual stimuli to assign cells as ON, OFF, or ON-OFF (Figure 2), (2) analysis of the spike waveform to classify cells as excitatory or inhibitory (Figure 1B), and (3) correlation of recording channels to cortical depth to assign each cell to a specific layer (Figure 1C–E). Our motivation for classifying cells into types was that previous studies repeatedly show that different types of neurons (e.g., RGCs) have different sensitivities to electric stimulation (Im and Fried, 2015); previous studies that have looked at cortical responses to electric stimulation did not similarly classify individual neurons into specific cell types. In general, we found similar distributions of cell types and similar response properties (Table 1) to those from previous studies (Chen et al., 2006; Niell and Stryker, 2008). Cortical neurons of different types and from different layers are known to receive synaptic inputs from distinct combinations or (presynaptic) excitatory and inhibitory neurons cells, and, the response properties of the different cell types are shaped by the different inputs they receive (Niell, 2015). Such differences suggested that different cell types might each have a unique response to electric stimulation of the retina. It was therefore somewhat surprising that the different types of cortical neurons had mostly similar responses: a single burst of spiking (that persisted for 40–50 ms (Figure 3B,C); the burst has an onset latency of ∼10 ms although we cannot rule out the possibility of earlier spikes that were obfuscated by the artifact. The fact that responses were largely similar suggests that the response differences that arise between different types of RGCs are lost as the neural signal propagates from the retina to V1, at least for the stimulating conditions used here. We cannot rule out the possibility that response differences exist in the lateral direction (Halupka et al., 2017), e.g., beyond the region captured by our single penetrating electrode, and this will be interesting to explore in future studies. The spikes that occurred after the period of inhibition were thought to be the recovery of spontaneous spikes and not an additional phase of the electrically elicited response – this is because cells with a low spontaneous rate did not show spikes at the end of the inhibition period.

### V1 Responses Are Suppressed by Stimulation Rates ≥2 PPS

The responses of V1 neurons to electric stimulation were highly sensitive to the rate at which stimulation was delivered. Even at rates of 2 PPS, there was a loss of responsiveness after the first few pulses (Figure 6A–E), e.g., robust responses were elicited by the first few pulses in a train but responses stopped completely (no spiking) to subsequent pulses. At higher rates of stimulation, the loss of responsiveness occurred after fewer pulses. This loss of responsiveness was not entirely surprising given the inhibitory signal that persisted for several hundred milliseconds following each pulse (Figure 3B,C), i.e., the inhibitory signal from a previous pulse was likely still in effect when the next pulse was delivered. The fact that the first few pulses routinely elicited responses suggests that whatever the source of this inhibitory signal, it does not completely overwhelm the excitatory input arriving from the retina; the inhibitory effect appears to be additive however, and becomes dominant over time. A similar loss of responsiveness to repetitive stimulation has been reported in RGCs *in vitro* (referred to as desensitization) (Jensen and Rizzo, 2007; Freeman and Fried, 2011) although the difference in duration between the RGC (Fried et al., 2006; Im and Fried, 2016) and cortical inhibitory signals makes it unlikely that RGC desensitization was responsible for the decreased sensitivity of cortical neurons observed here. The stable RGC responses observed here for rates up to 5 PPS (Figure 5) is also consistent with RGC inhibition having little or no contribution to the inhibitory signal in cortical neurons. It is also worth noting that while some types of RGCs can have complex responses to repetitive stimulation (Im and Fried, 2016), such complexity was not observed in cortical neurons – a single, short burst followed by a loss of responsiveness after a few pulses was found in all cells tested. Given the difference in sensitivity to repetitive stimulation between retinal and cortical neurons, the loss in sensitivity observed in cortical neurons is likely to arise as the neural signal propagates from the retina to the cortex although our results do not pinpoint the precise location or mechanism.

Some of the loss seen for low rates of stimulation may be attributable to band- or low-pass filtering of the visual pathway. Ridder reported that the amplitude of the VEP began to decrease in response to full field visual stimuli delivered at rates of 3 Hz while the ERG responded to higher frequencies (Ridder and Nusinowitz, 2006). By measuring VEPs from dark-adapted and light-adapted retinas, Ridder also showed that the rod and cone pathways had different sensitivities; the temporal tuning function of dark-adapted VEPs more closely matches the sensitivity to repetitive pulsatile stimulation seen here, raising the possibility that rods (or other retinal neurons that subserve the rod pathway) are activated by the pulses used here. This would not be entirely surprising given the mouse retina is strongly rod-dominated (97% of all photo receptors) (Carter-Dawson and LaVail, 1979; Jeon et al., 1998). Additional studies have shown that the sensitivity to repetitive stimulation varies for different types of visual stimuli, e.g., VEP amplitudes in response to sinusoidal gratings remain consistent at rates up to 5 Hz (Porciatti et al., 1999). This suggests that stimuli that activate spatially confined regions of the retina may result in better responsivity to higher frequencies and thus small, implantable electrodes may have better temporal responsivity than the large extraocular electrodes used here. Similarly, stimuli that preferentially target the cone pathway may also have improved sensitivity to higher rates of stimulation. While the temporal dynamics of the mouse and primate retinas are quite different, clinical reports consistently describe a limitation in the rate at which stimuli can be effectively delivered (Perez Fornos et al., 2012) and thus, the ability to better control the spread of activation and/or the specific cell types activated may help to improve the temporal responsiveness of clinical devices.

It is important to note also that the long-duration stimuli used here (2 ms/phase) were designed to activate outer retinal neurons which in turn activate RGCs (referred to as indirect activation). In addition to producing one or more robust bursts of spiking, this approach also typically results in strong activation of inhibitory neurons that contributes to desensitization. Stimuli that activate RGCs directly (e.g., short-duration pulses), elicit only a single, short-latency spike per pulse. While direct activation can produce very high rates of spiking (Fried et al., 2006; Sekirnjak et al., 2006), we were not able to evaluate whether this approach could improve the temporal responsiveness of cortical neurons. This is because the electrical artifact from short-latency pulses blocked the short-latency responses that we were trying to measure (not shown). The ability to reliably remove the artifact would be of great help for pursuing this line of investigation in the future. This may be possible via the use of small electrodes implanted in the retina as they will greatly reduce the amplitude required for activation. Alternatively, other forms of activation can reduce or eliminate the artifact (Tomita et al., 2010; Nirenberg and Pandarinath, 2012; Lee et al., 2016; Lee and Fried, 2017), thereby allowing unobstructed visualization of all responses.

### Sinusoidal Stimulation Creates Temporal Offsets in ON vs. OFF Cells

While the timing of the responses to pulsatile electric stimulation were similar for both ON and OFF neurons in the visual cortex, the use of low frequency sinusoidal stimulation resulted in a temporal offset between the two (Figure 8). Consistent with earlier work in the rabbit (Twyford and Fried, 2016), we first showed that ON RGCs in the mouse responded strongly during the anodal phase of low-frequency sinusoidal stimulation while OFF RGCs remained quiet (Figure 8A–D). During the cathodal phase OFF cells responded strongly while ON cells were quiet. This temporal offset in response timing is thought to arise because photoreceptors are activated by long, slowly changing stimuli and thus the sign-inverting synapses between photoreceptors and ON bipolar cells alters the sensitivity to stimulus polarity (and timing) for the ON and OFF cell types. When we subsequently recorded from ON and OFF cells in visual cortex, we found a similar offset in phase: OFF cells responded during the cathodal phase of stimulation while ON cells responded during the anodal phase (Figure 8E–H). This result is encouraging because it not only indicates that the response timing is maintained as the electrically induced signal propagates from the retina to the visual cortex but also suggests that the use of stimulation strategies that better replicate physiological patterns of spiking in the retina may also lead to patterns of spiking in the cortex that better resemble natural physiology. It is tempting to speculate that because cortical activity better reflects psychophysical percepts (Salzman et al., 1990; Knierim and van Essen, 1992), the use of low-frequency sinusoidal stimulation might improve contrast sensitivity (or other elements of artificial vision). However, it is important to point out that earlier attempts to evoke more natural signaling patterns, e.g., the use of stochastic resonance with cochlear prostheses, did not always lead to improved clinical outcomes unless optimized parameters, such as the level of added noise to enhance detectability, or the information content of a signal (e.g., trains of action potentials) (Moss et al., 2004) were incorporated. Thus, the clinical benefits associated with better matches to retinal signaling, e.g., with low-frequency stimulation will need to be verified in future testing. Also, it is far from certain that strategies that target photoreceptors will be of use in the degenerate retina, although there is some evidence to suggest that parts of photoreceptors can still be harnessed for at least some forms of degeneration (Busskamp et al., 2010). An additional concern is that the low-frequency sinusoidal waveforms described here require a large amount of charge delivery and thus increase the potential for electrochemical damage to the electrode. Any potential benefit of this approach will need to be evaluated in light of this risk. Nevertheless, other stimulation strategies have been proposed to selectively target ON vs. OFF RGCs that do not require intact photoreceptors (Jepson et al., 2014b; Twyford et al., 2014). It will be interesting to learn whether these strategies similarly result in cortical activity that better matches physiological signaling, and if so, whether they ultimately improve the quality of elicited percepts.

## Author Contributions

SR and SF conceived and designed the study, analyzed the results, wrote the manuscript, and prepared the figures. SR performed the animal experiments except for the *in vitro* retinal experiments, which was performed by PW.

## Conflict of Interest Statement

The authors declare that the research was conducted in the absence of any commercial or financial relationships that could be construed as a potential conflict of interest.
